# A novel and stress adaptive alternative oxidase derived from alternative splicing of duplicated exon in oyster *Crassostrea virginica*

**DOI:** 10.1038/s41598-017-10976-w

**Published:** 2017-09-07

**Authors:** Ming Liu, Ximing Guo

**Affiliations:** Haskin Shellfish Research Laboratory, Department of Marine and Coastal Sciences, Rutgers University, 6959 Miller Avenue, Port Norris, NJ 08349 USA

## Abstract

Alternative oxidase (AOX) is a mitochondrial inner-membrane oxidase that accepts electrons directly from ubiquinol and reduces oxygen to water without involving cytochrome-linked electron transport chain. It is highly conserved in many non-vertebrate taxa and may protect cells against hypoxia and oxidative stress. We identified two AOX mRNAs in eastern oyster *Crassostrea virginica*, CvAOXA and CvAOXB, which differ by 170 bp but encode AOXs of the same size. Sequence analyses indicate that *CvAOX* has 10 exons with a tandem duplication of exon 10, and 3′ alternative splicing using either the first or second exon 10 produces the two variants CvAOXB or CvAOXA, respectively. The second exon 10 in CvAOXA is more conserved across taxa, while the first exon 10 in CvAOXB contains novel mutations surrounding key functional sites. Both variants are expressed in all organs with the expression of CvAOXA higher than that of CvAOXB under normal condition. Under stress by air exposure, CvAOXB showed significantly higher expression than CvAOXA and became the dominant variant. This is the first case of alternative splicing of duplicated exon in a mollusc that produces a novel variant adaptive to stress, highlighting genome’s versatility in generating diversity and phenotypic plasticity.

## Introduction

**Figure 1:**
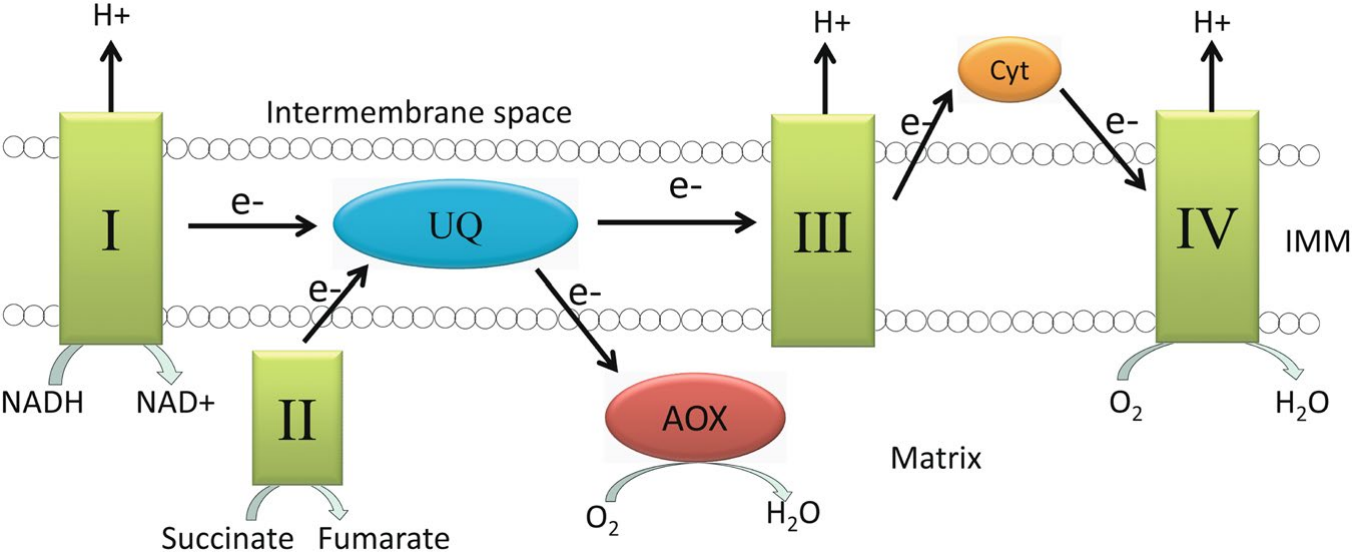
The respiratory electron transport chain of mitochondria showing the position of alternative oxidase (AOX), which introduces an alternative pathway at the point of ubiquinol (UQ). Complexes I, III and IV move protons across the inner mitochondrial membrane into the intermembrane space, producing a proton gradient which drives ATP synthesis.

The electron transport chain (ETC) is an essential part of cellular respiration where biochemical nutrients are converted to adenosine triphosphates (ATPs), the main energy source for cellular activities. In eukaryotes, ETC consists of a series of protein complexes that are located on the inner membrane of mitochondria. Electrons from donors such as nicotinamide adenine dinucleotide (NADH) and FADH2 are transferred through Complex I (NADH dehydrogenase) and II (succinate dehydrogenase) to ubiquinone, and then through Complex III to cytochrome c, which is subsequently oxidized by Complex IV (cytochrome c oxidase), reducing oxygen to water^1, 2^ (Fig. 1). Coupled with redox reactions in Complex I, III and IV, protons are transferred from the mitochondrial matrix to the inner membrane space creating a proton gradient that drives ATP synthesis. In most non-vertebrate taxa, an alternative pathway exists that takes electrons directly from the ubiquinone pool and reduces oxygen to water, bypassing complexes III, IV and cytochrome c and generating heat but few ATPs^3, 4^. The reaction is catalyzed by alternative oxidase (AOX)^3^, a diiron carboxylate protein^5^ located at the surface of the mitochondrial inner membrane.

AOX was first discovered in aroids^6^ and it was thought that heat generated by AOX activity was used by thermogenic plants to volatilize primary amines to attract pollinating insects^7, 8^. Subsequently AOX has been widely found in plants, fungi, protists and many non-vertebrate animal taxa suggesting that the alternative pathway may have more general and important functions^4, 9^. Besides thermogenesis, AOX in plants plays a critical role in responding to stress, balancing carbon metabolism and electron transport^9, 10^, regulating reactive oxygen species (ROS) generated by respiration^2, 11–13^, and resisting to potent inhibitors of the cytochrome pathway such as cyanide (CN)^14^, sulfide^15^, nitric oxide (NO)^16^, azide^17^, and antimycin A^18^. The function of AOX in animals are not well understood, although cyanide-resistant respiration has been observed in annelid worms *Nereis pelagica* and *Marenzelleria viridis* ^19, 20^ and molluscs *Arctica islandica* and *Geukensia demissa* ^20, 21^.

The eastern oyster *Crassostrea virginica* Gmelin 1791, is a bivalve mollusc native to Atlantic and Gulf Coasts of North America. It is an important fishery and aquaculture species as well as a keystone species for estuary ecology. As a filter-feeder that thrives in the intertidal zone, the eastern oyster can tolerate wide fluctuations in salinity, temperature and air exposure. Nevertheless, eastern oyster populations in the mid-Atlantic region have greatly declined due to overharvesting, diseases and environmental changes^22, 23^. Genomic research has been conducted to understand disease and stress resistance in oysters^24–27^. In analyzing transcriptome data, we identified two *AOX* mRNAs that differed by 170 bp. In this study, we studied the origin of the two *AOX* variants and their expression under air exposure. Here we report that these variants are produced by alternative splicing of a duplicated exon and may play a role in oyster’s adaptation to environmental stress.

## Results

### Characterization of CvAOX gene and protein

**Figure 2:**
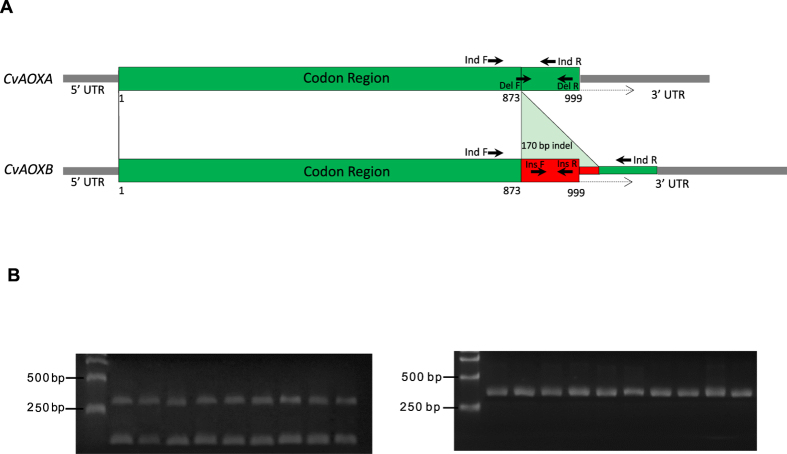
(**A**) Diagram of cDNAs corresponding to the two mRNA variants of *C. virginica* with the position of the indel, and the positions of primers used in this study. (**B**) PCR products from amplification of the indel using cDNA (left) and genomic DNA (right) as templates (the uncropped images are included in Supplementary Fig. S2). The expected size of the amplicon is 267 bp for the long variant and 97 bp for the short variant.

We identified seven *CvAOX* mRNAs by searching published and unpublished EST and transcriptome databases with *C. gigas AOX* sequence (CGI_10020743). Alignment of the seven sequences revealed a 170 bp indel with three sequences having the deletion (CvAOXA, short) and four sequences having the insertion (CvAOXB, long) (Fig. 2A). Open reading frame analysis indicated that the longer CvAOXB had a stop codon within the 170 bp insertion, producing a polypeptide of 332 amino acids, which is identical in length as AOX encoded by the shorter CvAOXA. Amplification of the indel region consistently produced two fragments from cDNA that differed by about 170 bp, but only one long fragment from genomic DNA (Fig. 2B), indicating that the two variants are not genomic mutations but produced through alternative splicing. Sequencing of the two mRNA fragments confirmed that they were identical except for the 170 bp indel.

**Figure 3:**
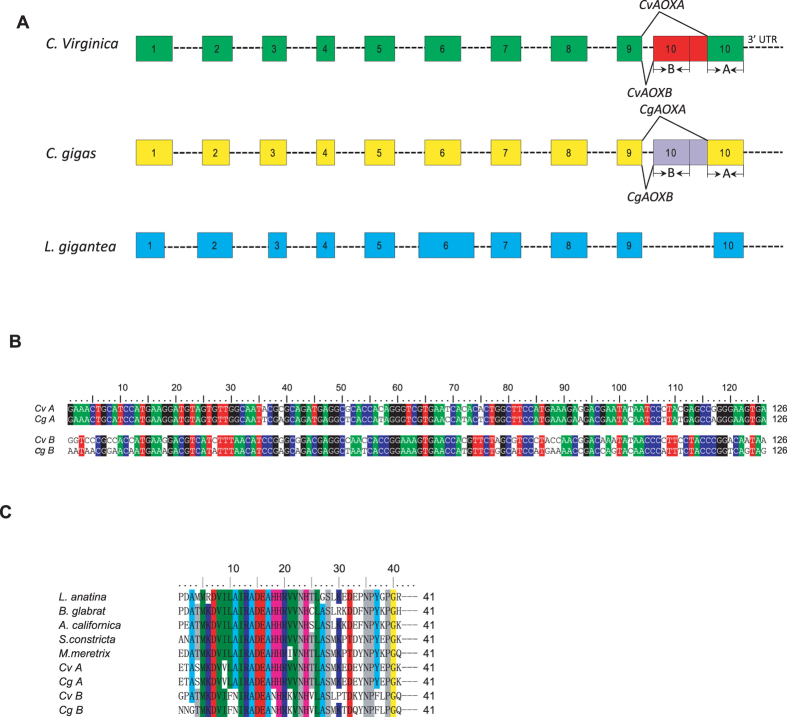
(**A**) Structure of *C. virginica, C. gigas* and *L. gigantea* AOX gene and mode of alternative splicing (alternative 3′ splicing site) in *C. virginica* and *C. gigas*. Blocks represent exons, dash represent introns;﻿ A and B are duplicated exon 10s. (**B**) Alignment of nucleotide sequences of the duplicated exon 10s in *C. virginica* (*Cv*) and *C. gigas* (*Cg*). (**C**) Alignment of peptide sequences of duplicated exon 10s in *C. virginica* and *C. gigas*, and exon 10s of other molluscs.

To identify intron-exon structure and confirm alternative splicing, we sequenced the gene body of *CvAOX*. Like known *AOXs* of other molluscs, *CvAOX* consisted of 10 exons and 9 introns (Fig. 3A). However, *CvAOX* contained a tandem duplication of exon 10 and part of the 3′ UTR, and an alternative 3′ splicing site that produces CvAOXA and CvAOXB by incorporating either the second exon 10 or both duplicated exons, respectively. The duplicated exon 10s showed significant homology in DNA (e-value = 9e^−14^) and protein (e-value = 4e^−20^) sequences. Because the duplicated exon 10s were terminal and contain stop codons, CvAOXB and CvAOXA encoded AOXs of the same size (332 aa), despite their difference in length.

We examined available genome and transcriptome data from other species and found the same duplication and alternative splicing in *C. gigas* but not in *Lottia gigantea*, *Aplasia californica, Meretrix meretrix* and *Sinonovacula constricta*, suggesting these events may be unique to oysters. DNA sequences of the duplicated exon 10 s showed significant divergence. Similarity between the two duplicated copies of exon 10 was 63.5% in *C. virginica* and 65.9% in *C. gigas*. Between the two species, the second exon 10 used by AOXA was more conversed (similarity = 90.5%) than the first exon 10 used by AOXB (similarity = 77.0%; Fig. 3B).

The 41-amino acid peptides coded by the duplicated exon 10s differed at 15 positions in *C. virginica* and at 14 positions in *C. gigas*. Peptide sequence coded by the second exon 10 used by CvAOXA was completely conserved between *C. gigas* and *C. virginica*, while peptides coded by the first exon 10 used by CvAOXB differed at 6 positions between the two species (Fig. 3C). Further, alignment of these 41 amino acids with that from other molluscs showed that the peptide coded by the second exon 10 was more similar with that of other species than that of the first exon 10 (Fig. 3C). Compared with that of two other bivalves, clams *Sinonovacula constricta* and *Meretrix meretrix*, the peptide coded by the second exon 10 differed by 7 amino acids while that coded by the first exon 10 differed by 14 amino acids. If only the 33 highly conserved amino acid positions were considered, the peptide coded by the second exon 10 diverged at 2 amino acid positions while that coded by the first exon 10 diverged at 8 positions, and the difference is significant (*p* = 0.039). These findings suggest that, after duplication, the second exon is completely conserved in protein sequences between the two oyster species, while the first exon 10 went through significant divergence probably because of relaxed selection pressure from infrequent usage. Further analysis revealed a small Ka/Ks value (0.04) between AOX sequences of the two oyster species, suggesting that the gene is under strong purifying selection.

**Figure 4:**
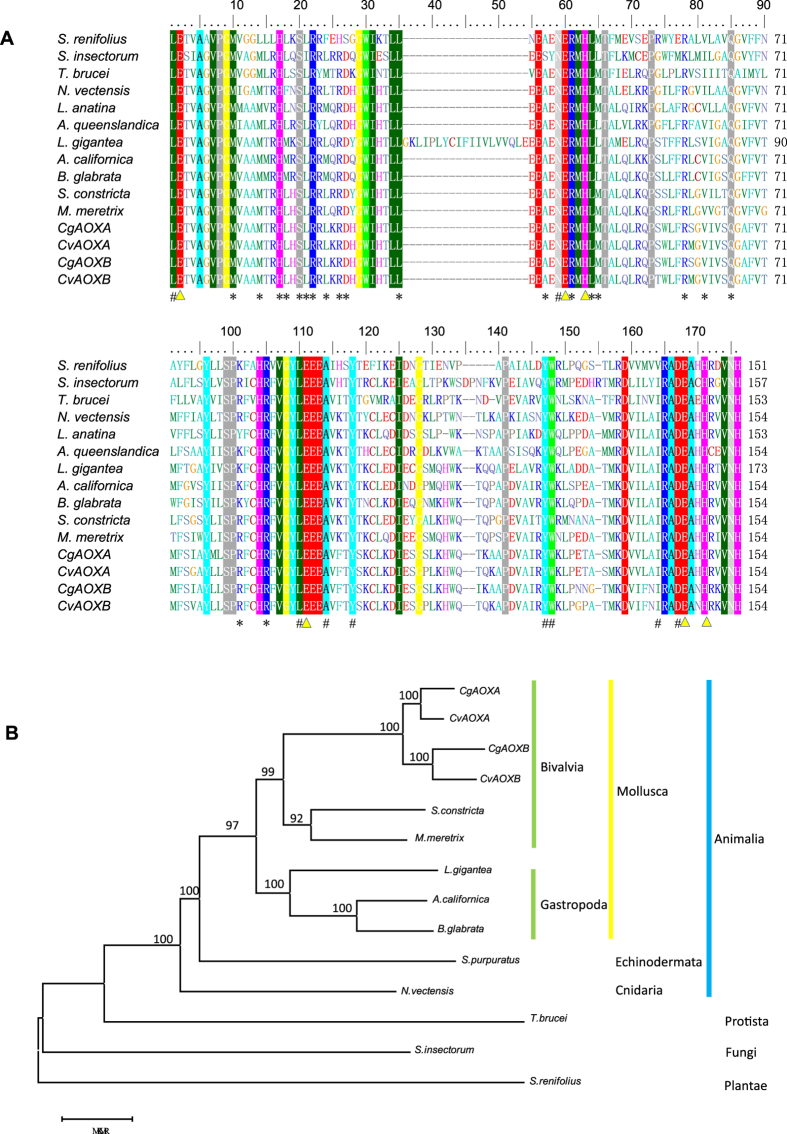
(**A**) Alignment of AOXs of *C. virginica*, *C. gigas* and 10 other species showing conservation of functional sites. Triangles represent the diiron binding motifs, # indicates the probable sites associated with activities of AOX, and asterisks are sites involved in interaction between monomers^55^. (**B**) Phylogenetic relationships among AOXs from *C. virginica*, *C. gigas* and 10 other species.

Sequence alignment of the AOX domain from ten species representing Protista, Fungi, Plantae, and Animalia, showed that key functional sites were complete conserved among species and between the two variants, although AOXB had several novel amino acids next to the functional sites that may affect site activity (Fig. 4A). Phylogenetic analysis of the entire AOX protein revealed a clade of molluscs and a closer relationship between the two oyster species, rather than between the two duplicated exons within species (Fig. 4B).

### Expression of CvAOX under air exposure

**Figure 5:**
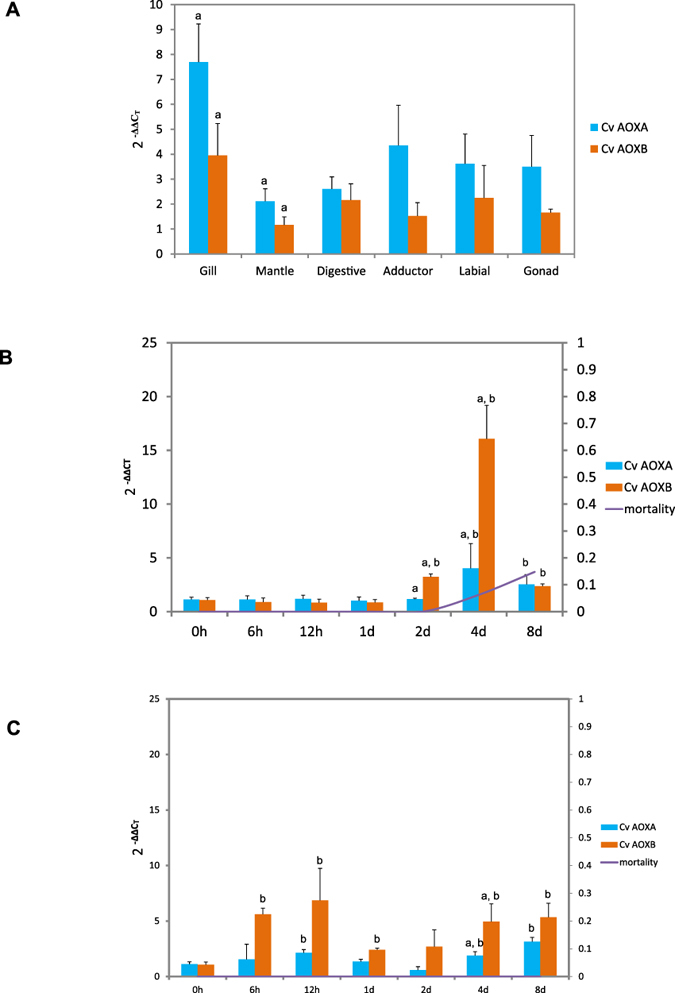
(**A**) Expression profile of CvAOXA and CvAOXB in different organs of *C. virginica*. Relative expression (y-axis) is calculated using 2^−ΔΔCт^ standardized by the lowest expression of CvAOXB in mantle. Error bars are standard errors. (**B**) Relative expression of CvAOXA and CvAOXB in gill under air exposure at 25 °C. (**C**) Relative expression of CvAOXA and CvAOXB in gill under air exposure at 5 °C. Expression levels of control samples (in water) in each time set are used as references. Lines and the secondary axis (right) show the mortality. Letter a designates significant difference between CvAOXA and CvAOXB. Letter b designates significant difference between challenged and control groups. Significance level is set at *p* < 0.05.

To determine whether AOX is involved in stress response in *C. virginica* and if the two variants respond differently, we measured mRNA expression of the two variants in oysters exposed to air exposure. Under normal conditions, both CvAOXA and CvAOXB were expressed in all six organs examined in this study: gill, mantle, digestive, adductor (striated), labial and gonad. The level of expression varied among organs, and the highest expression for both variants was found in gill, although the differences among most organs were not significant (Fig. 5A). In all organs, the expression level of CvAOXA was consistently higher than that of CvAOXB under normal conditions, and the difference was significant in gill (*p* = 0.014) and mantle (*p* = 0.049, Fig. 5A). When all organs were considered together, paired t-test showed that the expression of CvAOXA was significantly (*p* = 6.52E-06) higher than that of CvAOXB.

The expression of both CvAOXA and CvAOXB was up-regulated by prolonged air exposure. At 25 °C, CvAOXB showed significantly higher expression than CvAOXA at Day 2 (*p* = 0.005) and Day 4 (*p* = 0.043) after air exposure (Fig. 5B). At Day 4, the expression of CvAOXB was up-regulated by 16 folds, while the expression of CvAOXA was up-regulated by 4 folds (Fig. 5B). The up-regulation of both variants was reduced at Day 8 when oysters began to die. At 5 °C, CvAOXB was significantly up-regulated at 6 h, 12 h, 1 day (d), 4 d and 8 d after air exposure, and CvAOXA was up-regulated at 12 h, 4 d and 8 d after air exposure (Fig. 5C). The expression of CvAOXB was consistently higher than that of CvAOXA, although the difference was only significant (*p* = 0.014) at Day 4. When all oysters under air exposure were considered together regardless of exposure time, expression of CvAOXB was significantly (*p* = 0.001) higher than that of CvAOXA. These findings suggest that CvAOXA is preferentially expressed under normal conditions (Fig. 5A), while CvAOXB is preferentially expressed under stress by prolonged air exposure.

## Discussion

In plants, AOX is highly up-regulated when plants are exposed to stresses including temperature changes, drought, saline, hypoxia and infection by pathogens^28–32^. The up-regulation of *AOX* expression alleviates oxidative stress and maintains metablolic homeostasis in plants under stress^33, 34^. Two subfamilies of AOX exist in plants: AOX1 that has been found in all angiosperms examined to date and mainly functions in stress response, and AOX2 appears limited to dicot species where its physiological significance remains to be determined. For AOX1 subfamily, there are more than one gene loci found in many monocots such as rice *Oryza sativa* and wheat *Triticum aestivum* ^35^. Likewise, multiple AOX genes are also observed in many fungi and protists^36–38^. The existence of multiple AOX genes in these organisms may indicate gene and function diversification. In contrast, no more than one AOX gene has been identified in animals so far, and AOXs from animals are highly conserved^38^. The finding of two variants of AOX mRNA showing a 170 bp indel is therefore unexpected and interesting.

Our results show that there is only one AOX gene in *C. virginica* genome, and the two variants of AOX are not from insertion-deletion of genomic DNA, but due to alternative splicing involving the duplicated terminal exon 10. The duplication of exon 10 is supported by high homology in DNA (e-value = 9e^−14^) and protein (e-value = 4e^−20^) sequences and the same coding length between the duplicated copies. Gene duplication is an important mechanism for the emergence of new genes and functions during evolution. Exon duplication is also common in eukaryotes, and about 10% of all genes in human, fly and worm contain tandemly duplicated exons^39^, which may be an underestimate because some duplicates of short exons may have diverged beyond recognition. Exon duplication is often associated with alternative splicing of the duplicated exons^40^. Alternative splicing is the primary mechanism through which the genome generates mRNA and protein diversity from a given coding repertoire. The vast majority of eukaryotic genes go through alternative splicing^41^. Alternative splicing plays important roles in regulating development, physiology and homeostatis. One of the most important functions of alternative splicing is to provide alternative programs in response to environmental stress. Alternative splicing in plants is often associated with stress-related genes^42^ and may also play a role in stress response in bivalve molluscs^43, 44^.

AOX is a diiron oxidase located at mitochondrial inner membrane that reduces O_2_ to water without involving the cytochrome c pathway of the ETC and producing additional ATPs^45–47^. Mitochondrial ETC is a significant source of ATPs as well as reactive oxygen species (ROS). Stress causes electron leakage from ETC and generates excess ROS that damage cellular components^48–50^. AOX may participate in stress response by scavenging ROS and maintaining homeostasis^51^. Transgenic plant cells lacking AOX display higher rates of mitochondrial ROS generation^11^. AOX activation in durum wheat mitochondria leads to a decrease in the rate of superoxide anion generation^52^. In a marine worm and bivalve, it is also suggested excess oxygen can be eliminated by the alternative parthway to minimize oxidative stress^20^. In this study, expression of both variants of AOX is up-regulated by air exposure, suggesting AOX is important for stress response in *C. viginica* also. However, the up-regulation of AOX declined at day 8 at the on-site of mortalities indicates that there is a point when AOX response is no longer sufficient. Low temperature stress generates ROS^53^ but also reduces respiratory and metabolic rates, which may explain why AOX has an early but mild response to air exposure at 5 °C.

While CvAOXA shows higher expression than CvAOXB under normal condition (Fig. 5A) and both variants are up-regulated by stress, the expression of CvAOXB is significantly higher that of CvAOXA under prolonged air exposure (Fig. 5B and C), suggesting CvAOXB is prefered in response to stress. It is not clear why CvAOXA is preferentially expressed under normal conditions while CvAOXB is preferentially expressed under stress. The function of AOX may depend on the presence of the diiron binding motif (E2, E60, H63, E111, E168, H171), tyrosine residues especially Y118 for catalytic reaction^9, 54^, sites associated with ubiquinol binding and oxidization (L1, N59, L110, A114, Y147, W148, I164, D167), and twenty residues (M10, M14, H17, L18, S20, L21, R22, L24, R26, D27, L35, A57, R61, L64, M65, R78, Q85, V86, R101, R105) for interactions between the two monomers^55^. Although protein sequences of the two variants differed due to the use of the alternative exon, all sites that are known to be critical to AOX’s function are completely conserved. CvAOXB has several novel residues surrounding the second EXXH motif of the diiron center, although it is unknown if and how these novel mutations affects the function of CvAOXB. Further studies on the structure, cellular location and catalytic activity of this novel CvAOXB variant may provide insights into molecular mechansims of AOX’s function. Nevertheless, our results suggest that the alternative splicing variant CvAOXB is probably adaptive and preferred by oysters under stress. It should be pointed out that air exposure represents multiple stressors that may include hypoxia, changes in salinity, temperature and physical state, and response to air exposure may differ from that to other stressors.

The significant divergence between CvAOXA and CvAOXB and the relatively low divergence of both variants between *C. virginica* and *C. gigas* suggest that the duplication event occurred long before the divergence of the two species some 83 mya^56^. The finding that the duplication is not found in gastropods and other bivalves suggests that the duplication may be specific to oysters or certain subclasses of Bivalvia. Further studies in other oysters, bivalves and molluscs may provide better estimates of when the duplication occurred. The fact that the peptide coded by the second exon 10 in CvAOXA is completely conserved between the two oysters, while the peptide coded by the first exon 10 in CvAOXB diverged at 6 amino acid positions indicates that the second exon 10 is more important and subjected to stronger purifying selection pressure. Exon duplication may have relaxed selection pressure and allowed rapid accumulation of mutations in the first exon 10 in CvAOXB that is only preferred under stress. This is also supported by the observation that the second exon 10 in CvAOXA is more conserved than the first exon 10 in CvAOXB compared with other bivalve species. The preferential expression of CvAOXB under air exposure indicates that alternative splicing and the use of CvAOXB variant is beneficial to oysters under stress. Alternative splicing is known to participate in stress response in bivalve molluscs^43, 44^, and this study further demonstrates for the first time in a mollusc that exon duplication and alternative splicing in combination create a novel variant that is adaptive to environmental stress. Alternative splicing of duplicated exons^40^ may be an important mechanism of generating protein diversity and responding to stress in molluscs, which deserves further investigation.

## Materials and Methods

### Characterization of *C. virginica* AOX (CvAOX)

CvAOX mRNA sequences were obtained by searching *Crassostrea gigas* AOX (*CgAOX*) cDNA sequence (GenBank: ACL31211) in published and unpublished transcriptome databases of *C. virginica*. *CvAOX* sequences were aligned using *Clustal W* ^57^ which revealed two variants differed by ~170 bp (Fig. 2A).

To verify if the size difference is due to an indel in genomic DNA or from alternative splicing, primers (Table 1, Fig. 2A) were designed to amplify the region in question using both cDNA and genomic DNA as templates. Genomic DNA and cDNA from gills of nine *C. virginica* as well as genomic DNA of 31 oysters from four wild populations: Rhode Island (8), Delaware Bay (8), Florida (7) and Texas (8), were extracted and used for amplification. Polymerase Chain Reaction (PCR) was carried out in 10 µl volume containing 20 ng of DNA, 1 × PCR buffer, 1.5 mM of MgCl_2_, 0.2 mM of dNTP, 200 nM of each primer and 0.2 U of GoTaq polymerase (all from Promega) using the following profile: 95 °C for 5 min; 35 cycles of 95 °C for 30 sec, 57 °C for 30 sec, and 72 °C for 45 sec; and a final extension at 72 °C for 10 min. PCR products were analyzed in 1% agarose gels.

**Table 1:** Primers used for amplification of AOX in *C. virginica*.

Primer name	Sequence 5′-3′	usage
Ind F	AAAACACTGGCAGACTCAGAAAGC	Amplify the indel
Ind R	GCGTATTGCCAACACTACATCC	Amplify the indel
Del F	GCTACTGGAAACTTCCGGAAA	RT-PCR for CvAOXA
Del R	CATGGAAGCCAGTGTGTGATTC	RT-PCR for CvAOXA
Ins F	AAGGACGTCATCTTTAACATCC	RT-PCR for CvAOXB
Ins R	GTAGGAAGGGGTTATATTTGTC	RT-PCR for CvAOXB
G1 F	GGGAAGTTTACGACAAATTACG	Amplify the genomic DNA coding sequence
G1 R	ACATTATGGAGTTCTTCCTCTGAC	Amplify the genomic DNA coding sequence
G2 F	CACAGAGACACTTGTGGAGTCGCT	Amplify the genomic DNA coding sequence
G2 R	TTTCTCTGATCTTTCACCCCAGTTG	Amplify the genomic DNA coding sequence
G3 F	CTCCAGAGGGCTTTGTTGACAAG	Amplify the genomic DNA coding sequence
G3 R	CAGTCTCCAGGAAACAGATTCGT	Amplify the genomic DNA coding sequence
G4 F	CTTGCCTTCCGATCTGTGAAG	Amplify the genomic DNA coding sequence
G4 R	GACGCCCATTCTGAATAACCAAG	Amplify the genomic DNA coding sequence
G5 F	CTCAAACGAGACCACGGATGGAT	Amplify the genomic DNA coding sequence
G5 R	AGTTTCCAGTAGCGGATAGCCACG	Amplify the genomic DNA coding sequence
G6 F	CTTTACCTACTCCAAGTGTTTGAAG	Amplify the genomic DNA coding sequence
G6 R	GTGTCCTTAGTTTCCGGCTGTT	Amplify the genomic DNA coding sequence
EF-1 F	ATCAACTTCCACTGGCCATC	Reference gene for RT-PCR
EF-1 R	TTTTCCCATCTCAGCTGCTT	Reference gene for RT-PCR

To determine gene intron-exon structure, primers were designed to amplify genomic sequence of *CvAOX* based on cDNA sequences (Table 1). PCR was carried out as described above using genomic DNA and the following profile: 95 °C for 5 min; 35 cycles of 95 °C for 45 sec, 56 °C for 45 sec, and 72 °C for 90 sec; and a final extension at 72 °C for 10 min. PCR products were purified and sequenced at GenScript Inc., USA. Sequences were assembled using SeqMan (DNASTAR Inc. http://www.dnastar.com). Introns were identified by sequence alignment and the GT-AG rule. Ten protein sequences of AOX representing Protista, Fungi, Plantae, and Animalia were obtained from NCBI (Table 2) and aligned with the protein sequences of CvAOX and CgAOX. Active and functional sites in the protein sequence were determined according to Shiba *et al*. 2013^55^. Phylogenetic tree was constructed with Bayesian model using the Markov Chain Monte Carlo (MCMC) approach^58^. The parameters for MCMC sampling were: generations = 2,000,000 (standard deviation of split frequencies was < 0.01) with sample frequency = 1000, chains = 4, and burn-in value = 250 (corresponding to 25% of the samples). The method was implemented in MrBayes 3.1^58^.

**Table 2:** Species and AOX sequences used for phylogenetic analysis.

Kingdom	Plylum	Species	Genbank no	Reference
**Protista**	Euglenozoa	*Trypanosoma brucei*	XP_822944	Berriman *et al*.^61^
**Fungi**	Ascomycota	*Sporothrix insectorum*	OAA58516	Shang *et al*.^62^
**Plantae**	Angiosperms	*Symplocarpus renifolius*	BAD83866	Onda *et al*.^63^
**Animalia**	Cnidaria	*Nematostella vectensis*	XM_001635879	Putnam *et al*.^64^
	Echinodermata	*Strongylocentrotus purpuratus*	XP_011669099	Sodergren *et al*.^65^
	Mollusca	*Aplysia californica*	XP_005099181	Lowe and Eddy^66^
		*Biomphalaria glabrata*	XP_013086260	Yoshino *et al*.^67^
		*Lottia gigantea*	XP_009054933	Simakov *et al*.^68^
		*Sinonovacula constricta*		Dong *et al*., unpublished
		*Meretrix meretrix*		Dong *et al*., unpublished
		*Crassostrea gigas AOXA*	NP_001292289	Medeiros *et al*.^69^
		*Crassostrea gigas AOXB*	XP_011419856	Zhang *et al*.^60^
		*Crassostrea virginica AOXA*		This study
		*Crassostrea virginica AOXB*		This study

### Expression of two CvAOX mRNA variants under air exposure

Adult eastern oysters (n = 436) from the Rutgers NEH^TM^ strain were used to study the expression of *CvAOX* under air exposure stress. To determine the basic transcription profile of *CvAOX*, six different organs (gill, mantle, digestive, striated adductor muscle, labial and gonad) of four oysters were collected and fixed in RNA*later* (Life Technologies) before the challenge experiment. The remaining oysters were equally divided into three groups. Oysters in two groups were subjected to air exposure at 25 °C and 5 °C. Oysters in the third group were kept in seawater at 25 °C as control. Each group was further divided into four replicates. Three organs (gill, mantle and digestive) from four oysters were sampled from each group and stored in RNA*later* at 6 h, 12 h, 1 day (d), 2 d, 4 d, and 8 d after exposure. RNA was extracted using RNeasy Mini Kit (Qiagen) and treated with DNase I (Qiagen) to prevent DNA contamination. First-strand cDNA was synthesized from 1 µg of total RNA using oligo dT, random 6 mers and reverse transcriptase in a volume of 25 µl using the PrimeScript™ RT reagent Kit (Perfect Real Time, Takara) following supplied protocol. Single-strand cDNA was used directly for real-time RT-PCR.

For expression analysis, two pairs of primers were designed to specifically amplify the two variants (Table 1, Fig. 2). Products for the two variants were verified by sequencing. Real-time quantitative RT-PCR was performed on an ABI 7500 Fast Real-Time PCR System (Applied Biosystems). We tested three housekeeping genes, *beta-actin, elongation factor 1 (EF-1)* and *18S* ^59^ and checked the expression profile of *EF-1*, *beta-actin* and *Glyceraldehyde 3-phosphate dehydrogenase (GAPDH)* under air exposure in *C. gigas* transcriptome data^60^. EF-1 exhibited the most stable expression and was used as the reference gene for this study. Quantitative RT-PCR was carried out in biological triplicate, each in a volume of 20 ml containing 10 μl of 2X Power SYBR Green PCR mater mix (Applied Biosystems), 1 μl cDNA, 0.8 μl each of 5 µM primer and 7.4 μl of deionized water. The amplification was programmed as: 2 min at 50 °C, 2 min at 95 °C, and 40 cycles at 95 °C for 15 s and 60 °C for 1 min with the fluorescent signal collection. Expression of CvAOXA and CvAOXB was measured using the 2^−ΔΔCt^ method, whereas Ct is threshold cycle number determined by the real-time PCR system and reflecting the concentration of target gene in the reaction, ΔCt is the difference in Ct between target and reference gene which provides a relative quantification for the target gene in each sample, and ΔΔCt is the difference that ΔCt of each challenged sample minus ΔCt of the control sample. The expression level was then calculated by 2^−ΔΔCt^ which represent an n-fold difference relative to the control. Differences between groups were tested with two-sample t-test and differences between CvAOXA and CvAOXB within the same oyster and organ were tested with paired t-test on the ΔCt values using GraphPad Prism version 5.00 (GraphPad Software).

## Electronic supplementary material

Supplementary Figures
