# Supplementary material for: A novel and stress adaptive alternative oxidase derived from alternative splicing of duplicated exon in oyster Crassostrea virginica
Source: Sci Rep. 2017 Sep 7;7:10785. doi: 10.1038/s41598-017-10976-w (PMC5589949; doi:10.1038/s41598-017-10976-w)

Supplementary Information

A novel and stress adaptive alternative oxidase derived from alternative splicing of duplicated exon in oyster *Crassostrea virginica*

Ming Liu and Ximing Guo*

Haskin Shellfish Research Laboratory, Department of Marine and Coastal Sciences, Rutgers University, 6959 Miller Avenue, Port Norris, NJ 08349, USA.

*Corresponding author: xguo@hsrl.rutgers.edu; 856-785-0074 (phone).

Figure S1 cDNA and protein sequences of two variants of CvAOX with primers highlighted. Primer sequences are provided in Table 1.

CvAOXA

CATGTGCGCTTGCGTCTTGGGGTTATTAAAGGGATTTACGTGCATTCAAGGATATTTAAGTGGGAAACATTTGAATAAGTAAAAATATTACATGTCTAGTGAGGAAATCTGTGTACTATAAAGTAAAACCTACAGTGGATCGTAGCAAAT

ATGGGAAGTTTACGACAAATTACGAGATTAAGTGAAAATTGTGTCAGGATTTTTTGTAAACAAATGAAGGATGCGGAAAATGCTTCAATTCTACTGAGAATAAATGGAATTCGCACTGGTAGTGGGTTGCGCACCGCTGCAACAAAGGCT
M G S L R Q I T R L S E N C V R I F C K Q M K D A E N A S I L L R I N G I R T G S G L R T A A T K A

GATGTTGATGAAAATATTAAGAAATTCAAAGAAGGAAATTTCGAAAAAATCCCAGACCCAAATCACCTGGATCATTTTAGAAAGTCGCAGAGCACAGAGACACTTGTGGAGTCGCTCAAGGACCCTCCTCCTATGGGCACACACACCCTC
D V D E N I K K F K E G N F E K I P D P N H L D H F R K S Q S T E T L V E S L K D P P P M G T H T L

CCACATCCGATATGGTCAGAGGAAGAACTCCATAATGTAAAAGTCACCCACAAGCCTCCAGAGGGCTTTGTTGACAAGCTTGCCTTCCGATCTGTGAAGCTGTTGAGATCAACGTTTGATTTGTTAACAGGATTCAACTGGGGTGAAAGA
P H P I W S E E E L H N V K V T H K P P E G F V D K L A F R S V K L L R S T F D L L T G F N W G E R

TCAAAGAAAAAATGGGTCTTACGAATCTGTTTCCTGGAGACTGTTGCTGGGGTACCCGGAATGGTTGCAGCGATGACGCGACATTTACACTCACTGCGCAGGCTCAAACGAGACCACGGATGGATACACACACTCCTAGAGGAAGCAGAA
S K K K W V L R I C F L E T V A G V P G M V A A M T R H L H S L R R L K R D H G W I H T L L E E A E

AACGAGCGGATGCACTTAATGACGGCCTTACAGCTGCGTCAGCCATCTTGGTTATTCAGAATGGGCGTCATCGTGTCTCAAGGGGCGTTTGTGACAATGTTCAGTGGCGCGTACCTGCTTAGCCCCCGTTTCTGCCATCGCTTTGTCGGG
N E R M H L M T A L Q L R Q P S W L F R M G V I V S Q G A F V T M F S G A Y L L S P R F C H R F V G

Ind F → Del F →

TACCTAGAGGAGGAAGCAGTCTTTACCTACTCCAAGTGTTTGAAGGATATAGAATCCGGTCCCTTAAAACACTGGCAGACTCAGAAAGCCCCGGACGTGGCTATCCGCTACTGGAAACTTCCGGAAACTGCATCCATGAAGGATGTAGTG
Y L E E E A V F T Y S K C L K D I E S G P L K H W Q T Q K A P D V A I R Y W K L P E T A S M K D V V

← Ind R ← Del R

TTGGCAATACGCGCAGATGAGGCGCACCACAGGGTCGTGAATCACACACTGGCTTCCATGAAAGAGGACGAATATAATCCCTACGAGCCGGGGAAGTGAACAGCCGGAAACTAAGGACACTGAAGGGTCATTAACGCTATAGTTTATGCT
L A I R A D E A H H R V V N H T L A S M K E D E Y N P Y E P G K *

Exon 10

TGTTGACCAATGTTTGGTCTTACGTTCTTTCAGAATTTGCATACCAGACATTGTGTACTGTTTTGTTACACGTGTTAATGTTAAATACCGCCGACATGACGTAAAACATCAAACAATCAATCAATCTTTACACGTTTTAAGTGATGCATT

GATATTAAACTGTATTTCCGCCCGAATTTTGTGAACATTTCATTTGAATTTGTTACGCTAGAAGATCATTTGAACACCGTGGCTTGCATTTCATAGACTGAATCAGTAAATGTCATTTGTGATGCCATGTATTTCTATACAATTTTGTAG

TAAACGTGGAAAGAAAATCATACCGGATATGTATGATTGAGTTATTGTCATGTGTGTTTTGAATTGATGTGACTCCTGGATGAAATATGTATTGTAAAGTTTTGTTTTACAAAACTTCTGTTTAAAAACATTGAATTTTATTTGTAAAAT

GTTT

CvAOXB

CATGTGCGCTTGCGTCTTGGGGTTATTAAAGGGATTTACGTGCATTCAAGGATATTTAAGTGGGAAACATTTGAATAAGTAAAAATATTACATGTCTAGTGAGGAAATCTGTGTACTATAAAGTAAAACCTACAGTGGATCGTAGCAAAT

ATGGGAAGTTTACGACAAATTACGAGATTAAGTGAAAATTGTGTCAGGATTTTTTGTAAACAAATGAAGGATGCGGAAAATGCTTCAATTCTACTGAGAATAAATGGAATTCGCACTGGTAGCGGGTTGCGCACCGCTGCAACAAAGGCT
M G S L R Q I T R L S E N C V R I F C K Q M K D A E N A S I L L R I N G I R T G S G L R T A A T K A

GATGTTGATGAAAATATTAAGAAATTCAAAGAAGGAAATTTCGAAAAAATCCCAGACCCAAATCACCTGGATCATTTTAGAAAGTCGCAGAGCACAGAGACACTTGTGGAGTCGCTCAAGGACCCTCCTCCTATGGGCACACACACCCTC
D V D E N I K K F K E G N F E K I P D P N H L D H F R K S Q S T E T L V E S L K D P P P M G T H T L

CCACATCCGATATGGTCAGAGGAAGAACTCCATAATGTAAAAGTCACCCACAAGCCTCCAGAGGGCTTTGTTGACAAGCTTGCCTTCCGATCTGTGAAGCTGTTGAGATCAACGTTTGATTTGTTAACAGGATTCAACTGGGGTGAAAGA
P H P I W S E E E L H N V K V T H K P P E G F V D K L A F R S V K L L R S T F D L L T G F N W G E R

TCAGAGAAAAAATGGGTCTTACGAATCTGTTTCCTGGAGACTGTTGCTGGGGTACCCGGAATGGTTGCAGCGATGACGCGACATTTACACTCACTGCGCAGGCTCAAACGAGACCACGGATGGATACACACACTACTAGAGGAAGCAGAA
S E K K W V L R I C F L E T V A G V P G M V A A M T R H L H S L R R L K R D H G W I H T L L E E A E

AACGAGCGGATGCACTTGATGACCGCCTTACAGCTGCGTCAGCCAACTTGGTTATTCAGAATGGGCGTCATCGTGTCTCAAGGGGCGTTTGTGACAATGTTCAGTGTCGCGTACCTGCTTAGCCCCCGTTTCTGCCATCGCTTTGTCGGG
N E R M H L M T A L Q L R Q P T W L F R M G V I V S Q G A F V T M F S V A Y L L S P R F C H R F V G

Ind F → Ins F →

TACCTAGAGGAGGAAGCAGTCTTTACCTACTCCAAGTGTTTGAAGGATATAGAATCCGGTCCCTTAAAACACTGGCAGACTCAGAAAGCTCCGGACGTGGCTATCCGCTACTGGAAACTTCCGGGTCCCGCCACCATGAAGGACGTCATC
Y L E E E A V F T Y S K C L K D I E S G P L K H W Q T Q K A P D V A I R Y W K L P G P A T M K D V I

← Ind R

TTTAACATCCGGGCGGACGAGGCCAACCACCGGAAAGTGAACCACGTTCTGGCGTCCCTACCAACCGACAAATATAACCCCTTCCTACCTGGTCAATAACAAACACACAGACCCTAAATGTGCCTCATCTCTGTCTTTTTCAGGAAACTG
F N I R A D E A N H R K V N H V L A S L P T D K Y N P F L P G Q *

← Ind R **Exon 10**

CATCCATGAAGGATGTAGTGTTGGCAATACGCGCAGATGAGGCGCACCACAGGGTCGTGAATCACACACTGGCTTCCATGAAAGAGGACGAATATAATCCCTACGAGCCGGGGAAGTGAACAGCCGGAAACTAAGGACACTGAAGGGTCA

TTAACGCTATAGTTTATGCTTGTTGACCAATGTTTGGTCTTACGTTCTTTCAGAATTTGCATACCAGACATTGTGTACTGTTTTGTTACACGTGTTAATGTTAAATACCGCCGACATGACGTAAAACATCAAACAATCAATCAATCTTTA

CACGTTTTAAGTGATGCATTGATATTAAACTGTATTTCCGCCCGAATTTTGTGAACATTTCATTTGAATTTGTTACGCTAGAAGATCATTTGAACACCGTGGCTTGCATTTCATAGACTGAATCAGTAAATGTCATTTGTGATGCCATGT

ATTTCTATACAATTTTGTAGTAAACGTGGAAAGAAAATCATACCGGATATGTATGATTGAGTTATTGTCATGTGTGTTTTGAATTGATGTGACTCCTGGATGAAATATGTATTGTAAAGTTTTGTTTT

Figure S2 The uncropped images of electrophoretic gels of all the PCR products. Both genomic DNA and cDNA of *C. virginca* were amplified using Primer Ind F and Ind R to detect the indel. Genomic DNA templates are from four wild populations: Rhode Island (RI), Delaware Bay (DB), Florida (FL) and Texas (TX). Nine cDNA templates are from the oyster samples used in air exposure challenging (Time 0, gill). The expected size of the amplicon in cDNA is 267 bp for the long variant and 97 bp for the short variant.

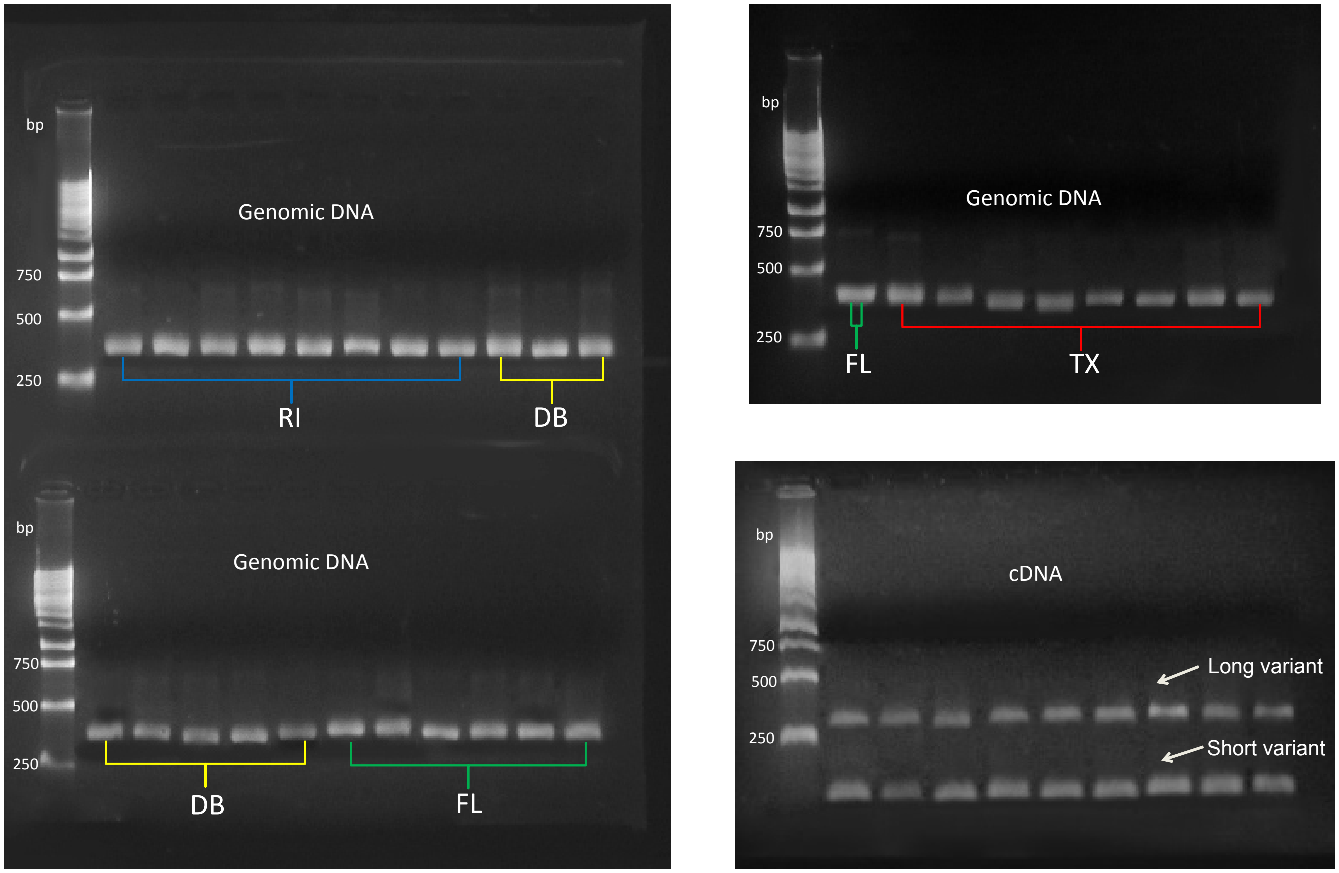

Supplement: Supplementary file 1 — Supplementary Figures [file 41598_2017_10976_MOESM1_ESM.pdf]
